# Complete Genome Sequence of Klebsiella oxytoca Strain AHC-6, Isolated from a Patient during Acute Antibiotic-Associated Hemorrhagic Colitis

**DOI:** 10.1128/mra.01350-22

**Published:** 2023-03-16

**Authors:** Johanna Dabernig-Heinz, Gabriel E. Wagner, Eva Leitner, Werner Ruppitsch, Ivo Steinmetz, Christoph Högenauer, Ellen L. Zechner, Sabine Kienesberger

**Affiliations:** a Diagnostic and Research Institute of Hygiene, Microbiology, and Environmental Medicine, Medical University of Graz, Graz, Austria; b Institute of Medical Microbiology and Hygiene, Austrian Agency for Health and Food Safety, Vienna, Austria; c Division of Gastroenterology and Hepatology, Department of Internal Medicine, Medical University of Graz, Graz, Austria; d Institute of Molecular Biosciences, University of Graz, Graz, Austria; e BioTechMed-Graz, Graz, Austria; f Field of Excellence BioHealth, University of Graz, Graz, Austria; University of Rochester School of Medicine and Dentistry

## Abstract

Klebsiella oxytoca is a ubiquitous bacterium that is increasingly associated with inflammatory diseases. Here, we report the hybrid assembled genome for cytotoxic K. oxytoca strain AHC-6. The genome comprises a total of 5.7 Mbp, with a GC content of 55.2% and 5,258 coding sequences after assembly and annotation.

## ANNOUNCEMENT

Klebsiella oxytoca *sensu lato* strains are ubiquitous Gram-negative bacteria. The K. oxytoca species complex (KoSC) consists of at least six very closely related species (1). KoSC members are early colonizers of the infant gut (2–6). K. oxytoca, Klebsiella grimontii, Klebsiella pasteurii, and Klebsiella michiganensis frequently encode the capacity for biosynthesis of the enterotoxins tilimycin and tilivalline in the tilimycin/tilivalline (*til*) gene cluster (7–10), alternatively called the kleboxymycin biosynthesis gene cluster (11). Cytotoxic KoSC members are causative agents of antibiotic-associated hemorrhagic colitis (AAHC) (12) and are associated with necrotizing enterocolitis (NEC) in premature infants (13).

K. oxytoca strain AHC-6 was obtained from a patient during acute AAHC (12). The donor was recruited according to Medical University of Graz ethics approval (approval number 17-199 ex 05/06). AHC-6 was isolated on MacConkey agar, identified with the API 20E test (bioMérieux), and cultured in tryptic soy (TS) medium at 37°C, before passaged five times and stored at −80°C in 20% glycerol. Multilocus sequence type 1 was assigned (14) (isolate 34) and cytotoxicity towards cultured human cells was assessed (15) (see strain 04/1O). Murine infection models confirmed *in vivo* toxin production by AHC-6 (16, 17), identified the molecular targets of tilimycin and tilivalline, providing a mechanistic link to epithelial cell damage (7, 16), and showed that the genotoxicity induced by tilimycin generates DNA lesions in enterocytes (16) and decreases microbial diversity while promoting the emergence of antibiotic resistance in the gut microbiome (18).

Prior to DNA purification (NucleoSpin microbial DNA kit; Macherey-Nagel) for separate libraries, AHC-6 was thawed from the glycerol stock on TS agar, and a single colony was expanded and passaged twice. DNA extracts were additionally purified according to the AMPure XP beads protocol (Beckman Coulter). The native barcoding kit 24 (SQK-NBD112.24; Oxford Nanopore Technologies [ONT]) was used for library preparation, utilizing the long fragment buffer to enrich for DNA fragments of >3 kb. This library was sequenced with a R10.4 flow cell on a MinION Mk1B system (ONT). Paired-end 300-bp short reads were obtained by sequencing a Nextera XT (Illumina) library on an Illumina MiSeq system.

Default parameters were used for all software unless otherwise specified. Raw data were base called with Guppy v6.0.6 (ONT) in super-accurate mode (19), and reads were split with Duplex Tools v0.2.9 (ONT). For quality control, the base-called reads were filtered using Filtlong v0.2.1 (20) (minimum length 1000 base-pairs, keep_percent 90, and target bases 620000000). The genome was assembled using Flye assembler v2.9 (21) (nano-hq). Flye reported one circular contig. For the Racon polishing step, filtered reads were mapped back to the assembly with minimap2 v2.24 (22) (–ax map-ont) and polished with Racon v1.3.1 (23) (–m 8 –x −6 –g −8 –w 500). The assembly was polished using Medaka v1.5 (ONT) with model r104_e81_sup_g5015. For hybrid assembly, short reads were filtered with Trimmomatic v0.39 (24) (HEADCROP 10 and SLIDINGWINDOW 5:20). Filtered reads were used to polish the long-read assembly with POLCA (25) and Polypolish (26) (–v 0.6). Genome annotation utilized the NCBI Prokaryotic Genome Annotation Pipeline (PGAP) v6.0 (27) with manual correction.

Characteristics of the sequencing data and genome are summarized in Table 1.

**TABLE 1:** Characteristics of sequencing, assembly, and the final circular genome

Parameter	Finding
Total genome size (bp)	5,764,439 (circular)
No. of contigs	1
Filtered long-read coverage (×)	106
No. of raw long reads	53,186
Long-read fragment *N*_50_ (bp)	23,280
Short-read coverage (×)	212.7
No. of raw short reads	3,399,558
Short-read fragment size (bp)	300
GC content (%)	55.2
Total no. of genes	5,375
Total no. of coding sequences	5,258
No. of RNA genes	117
No. of complete rRNAs (5S, 16S, 23S)	9, 8, 8
No. of tRNAs	84
No. of noncoding RNAs	8
Total no. of pseudogenes	73

### Data availability.

The assembled genome was deposited in NCBI GenBank under BioProject accession number PRJNA847041 and BioSample accession number SAMN28911479. Raw reads were submitted to the Sequence Read Archive (SRA); Illumina raw reads can be found under SRA accession number SRR21540817 and Nanopore raw reads under SRA accession number SRR21560188. The version described here is the first version of the assembly.
